# Investigation of Bovine Serum Albumin (BSA) Attachment onto Self-Assembled Monolayers (SAMs) Using Combinatorial Quartz Crystal Microbalance with Dissipation (QCM-D) and Spectroscopic Ellipsometry (SE)

**DOI:** 10.1371/journal.pone.0141282

**Published:** 2015-10-27

**Authors:** Hanh T. M. Phan, Shannon Bartelt-Hunt, Keith B. Rodenhausen, Mathias Schubert, Jason C. Bartz

**Affiliations:** 1 Department of Civil Engineering, University of Nebraska-Lincoln, Lincoln, Nebraska, United States of America; 2 Department of Electrical and Computer Engineering, University of Nebraska-Lincoln, Lincoln, Nebraska, United States of America; 3 Department of Medical Microbiology and Immunology, Creighton University, Omaha, Nebraska, United States of America; Martin-Luther-Universität Halle-Wittenberg, GERMANY

## Abstract

Understanding protein adsorption kinetics to surfaces is of importance for various environmental and biomedical applications. Adsorption of bovine serum albumin to various self-assembled monolayer surfaces including neutral and charged hydrophilic and hydrophobic surfaces was investigated using *in-situ* combinatorial quartz crystal microbalance with dissipation and spectroscopic ellipsometry. Adsorption of bovine serum albumin varied as a function of surface properties, bovine serum albumin concentration and pH value. Charged surfaces exhibited a greater quantity of bovine serum albumin adsorption, a larger bovine serum albumin layer thickness, and increased density of bovine serum albumin protein compared to neutral surfaces at neutral pH value. The quantity of adsorbed bovine serum albumin protein increased with increasing bovine serum albumin concentration. After equilibrium sorption was reached at pH 7.0, desorption of bovine serum albumin occurred when pH was lowered to 2.0, which is below the isoelectric point of bovine serum albumin. Our data provide further evidence that combinatorial quartz crystal microbalance with dissipation and spectroscopic ellipsometry is a sensitive analytical tool to evaluate attachment and detachment of adsorbed proteins in systems with environmental implications.

## Introduction

Protein adsorption onto surfaces plays a significant role in many fields including medicine, biology, pharmaceutical development and environmental engineering [1] with applications in protein—DNA interactions; drug formulation and storage; and environmental decontamination. Protein attachment processes have been investigated on a variety of natural and engineered surfaces [2–11]. Generally, protein adsorption at the solid/liquid interface can occur due to electrostatic interactions, hydrophobic interactions, and hydrogen-bonding interactions. This process is influenced by properties of the protein (such as stability), the adsorbent surface, and solution such as ionic strength and pH value [12]. Once a protein is associated with a surface, processes such as protein reorientation can induce conformational changes accompanied by protein unfolding, lateral protein-protein interactions, and desorption [2]. Structural changes of an adsorbed protein may alter protein biological function [13], but observation of structural changes of an adsorbed protein are challenging. We propose the use of quartz crystal microbalance with dissipation (QCM-D) combined with spectroscopic ellipsometry (SE) to allow for measurement of complementary information *in-situ* which allows simultaneous determination of adsorption thickness, adsorption mass, and porosity. QCM-D/SE is a technique with broad potential applications in characterizing biological phenomena on the nanoscale. This technique can provide insight into protein interactions at solid/liquid interfaces including structural arrangements, cooperative adsorption, cross-linking, adsorption kinetics, and protein aggregation [14–17].

Bovine serum albumin (BSA) is suitable for attachment studies because of its high stability, its availability at high purity and its water solubility [4, 18]. Solution pH affects BSA adsorption as the isoelectric point (IEP) of BSA is at pH 4.5–5.0, therefore the protein is negatively charged at neutral pH [3, 4, 7, 18–20], and positively charged under acidic conditions. The three domains of BSA with varying surface charge density influences BSA adsorption on charged surfaces [21, 22]. For instance, the occurrence of both negatively charged amino acids (glutamic acid, aspartic acid) and positively charged residues (lysine, histidine) on BSA can result in attachment to both positively and negatively charged surfaces [3, 18]. At pH values above the IEP of the protein, adsorption was observed on negatively charged surfaces due to electrostatic interactions with positively charged amino acid residues [3, 7]. In addition, BSA adsorption is a pH-dependent phenomenon, whereby maximum protein adsorption is observed near the IEP with decreasing adsorption observed at pH above or below the IEP [4, 6, 7].

BSA adsorbs to a variety of surfaces such as titanium powder [19], TiO_2_ [4], clays [3], polymers [7], and oxide minerals [6] as measured by spectrophotometric measurements [4], colorimetric estimation [7], and various spectroscopic techniques including NMR, fluorescence, circular dichroism, and FTIR-spectroscopy [3]. There are a limited number of studies of BSA adsorption on biofunctional and environmentally-relevant surfaces such as self-assembled monolayers (SAMs). A recent study analyzing DNA nanoparticle and fetal bovine serum (FBS) protein attachment to model biomaterial substrates was one of the first to evaluate protein adsorption using QCM-D/SE [15, 23].

The objective of this study was to use combinatorial *in-situ* QCM-D/SE to investigate the dynamic adsorption processes of BSA to various environmentally-relevant SAM surfaces. The selected SAM surfaces carried distinct terminal functional groups represent dominant functional groups in soil minerals and soil organic matter and are used in this study to represent environmentally relevant surfaces. We determined BSA physical adsorption characteristics of BSA as a function of SAM properties, solution pH, and BSA concentration. We hypothesize that the attachment of BSA is strongly dependent on surface properties of the SAM monolayer, including the surface charge and hydrophobicity. Previous BSA adsorption studies have mainly focused on kinetic measurements and BSA conformational changes after surface adsorption. The combinatorial *in-situ* QCM-D/SE technique provides an additional parameter, volume fraction of adsorbate or porosity, which is a valuable parameter in determining biological properties of organic mass. To date, limited studies have measured BSA adsorption onto various SAM surfaces and no previous studies have reported the volume fraction (porosity) of adsorbed BSA to SAMs, which can provide information about the conformation of surface-adsorbed BSA.

## Materials and Methods

### SAM preparation

SAMs investigated in this study include 11-Mercapto-1-undecanol (MUOH, 99%, Fisher Scientific), 11-Mercaptoundecanoic acid (MUA, 98%, Fisher Scientific), 1-Decanthiol (DT10, 99%, Fisher Scientific), and 11-Amino-1-undecanthiol hydrochloride (AUT, > 90%, Dojindo). MUOH, MUA, and AUT have hydrophilic hydroxyl (–OH), carboxyl (–COOH), and amine (–NH_2_) terminal functional groups, respectively, while DT10 has a hydrophobic methyl (–CH_3_) terminal functional group (Table 1). At the neutral pH conditions used in this study (pH 6.7), MUOH and DT10 are neutral while AUT is positively charged and MUA is negatively charged. These SAMs are well characterized and used extensively as model surfaces [24, 25]. n-Alkanethiols attach to Au surfaces by chemisorption at a thiol group to form close-packed SAMs, leaving the other end of function groups (–CH_3,_ –OH, –COOH, and –NH_2_) available to bind proteins [26]. The Au substrate was chosen because it is biocompatible and can be easily modified with SAM attachment by forming stable metal-sulfur bonds [18, 27, 28].

**Table 1:** Investigated SAMs.

Short name	Long name	Chemical formula	Features	Charge (at pH 6.7)
**MUOH**	11-Mercapto-1-undecanol	HSCH_2_(CH_2_)_9_CH_2_OH	Hydrophilic (-OH tail)	Neutral
**MUA**	11-Mercaptoundecanoic acid	HSCH_2_(CH_2_)_8_CH_2_COOH	Hydrophilic (-COOH tail)	-
**AUT**	11-Amino-1-undecanthiol, hydrochloride	HSCH_2_(CH_2_)_9_CH_2_NH_2_ HCl	Hydrophilic (-NH_2_ tail)	+
**DT10**	1-Decanethiol	HSCH_2_(CH_2_)_8_CH_3_	Hydrophobic (-CH_3_ tail)	Neutral

To prepare the SAM surface, 2 mM of each alkanethiol solution was prepared using filtered and degassed 200 proof ethanol (Fisher Scientific). Quartz crystal sensors coated with a 100 nm Au layer were used as substrates. The sensors were manufactured by Biolin Scientific and used as received.

The Au-coated sensors were first rinsed with copious amounts of acetone (Fisher Scientific) followed by 200 proof ethanol before forming the SAM by immersing the wafer in 20 mL of 2.0 mM alkanethiol solution in an amber bottle covered by Ar gas stream for at least 45 min (MUOH and MUA), 60 min (AUT), and 18 hr (DT10) at room temperature. The prepared SAM-coated Au sensors were then rinsed with 200 proof ethanol to ensure the removal of physically-absorbed thiol molecules. The SAM surfaces were dried under N_2_ gas.

### Contact angle measurements

The contact angle quantitatively describes the wettability of a surface [2]. The static contact angles of the clean Au surfaces before SAM deposition and Au surfaces after SAM deposition were measured. To examine the difference in contact angle due to the presence of a SAM, we used a micro-syringe to place a sessile water drop on the Au surface. Contact angles were quantified using a Ramé-Hart Imaging system (Ramé-Hart, Inc.) and ImageJ software.

### *Ex-situ* ellipsometry

*Ex-situ* ellipsometry measurements were made using an M-2000-VI Spectroscopic Ellipsometer (J.A.Woollam Co., Inc.) to evaluate the thickness of the SAM layer deposited on a Au surface. *Ex-situ* ellipsometry measurements on the Au surfaces were made at room temperature both before and after SAM chemisorption in the spectral range of 370–1640 nm and at multiple angles of incidence with respect to the substrate normal from 45^°^ to 75^°^ in 10^°^ increments. We used a two-layer substrate-SAM optical model. The SAM was modeled by a Cauchy layer, where the extinction coefficient *k* is necessarily 0 and where we assumed the index of refraction *n* to be 1.5. The optical constants of the Au surface were determined from the measurement taken before SAM chemisorption, while the Cauchy layer had a thickness of 0. The Cauchy layer thickness was then allowed to vary by the optical model as model-calculated data and the experimental data taken after SAM chemisorption were best-matched by the WVASE^32^ software package (J.A.Woollam Co., Inc.).

### BSA protein

The BSA stock solution was generated by dissolving BSA powder (Fisher Scientific) in DI H_2_O to a final concentration of 1.0 mg/mL. BSA is a globular protein with the approximate shape of a prolate spheroid of dimensions 4 nm x 4 nm x 14 nm in aqueous solution [29]. To investigate adsorption as a function of BSA concentration, a set of experiments was conducted using the AUT SAM with BSA solution concentrations of 1,000 μg/mL, 1.0 μg/mL, 0.5 μg/mL, and 0.1 μg/mL.

### *In-situ* combinatorial QCM-D/SE

BSA adsorption to SAM-coated surfaces was monitored in real-time using *in-situ* combinatorial QCM-D/SE. Details about data acquisition and comparison between QCM-D and SE are described in a prior study by two of the authors [30]. Briefly, for a porous organic adsorbate layer, QCM-D is sensitive to the presence of both attached adsorbate and ambient liquid that is coupled to the adsorbate. On the other hand, for porous, transparent adsorbate layers with thickness that is very small compared to the wavelength of probing light (i.e., on the order of 10 nm or less), SE is not sensitive to ambient liquid within the adsorbate layer. Thus, the adsorbate volumetric porosity (f_o,V_) may be found if the SE-determined adsorbate thickness parameter d_SE_ and the QCM-D-determined adsorbate thickness parameter d_QCMD_ are known. For the special case where the adsorbate and liquid densities are assumed equivalent (for simplicity), f_o,V_ is the ratio of d_SE_ to d_QCMD_ [30, 31].

The combinatorial QCM-D/SE instrumentation consists of an M-2000-V SE (J. A. Woollam Co., Inc.), which measures 512 wavelengths in the visible and near-infrared spectrum simultaneously (370–1000 nm), with a mounted E1 QCM-D (Biolin Scientific). The optical model for *in-situ* measurements was a three-layer substrate-SAM-BSA layer under DI H_2_O solution ambient. Similarly, the protein layer was modeled by a Cauchy layer assuming *n* = 1.5. If there is error in this assumption, that error will propagate to the SE thickness value. For organic materials that are powders under experimental conditions, including the –NH_2_ terminated alkanethiols, quantitatively finding the index of refraction is problematic. Furthermore, we are also assuming BSA to be an organic thin layer. Prior to the *in-situ* QCM-D/SE measurement, three *ex-situ* SE measurements were taken to develop the optical model. Starting values were taken from the two-layer optical model described above. First, the SAM-coated sample was measured under air at a 65^°^ angle of incidence with respect to the substrate normal to confirm the optical model starting values. Second, the sample was placed in a windowed liquid chamber that allows for QCM-D measurements and for probing light to enter and exit at a 65^°^ angle of incidence with respect to the substrate normal; an SE measurement was taken under air ambient. Wavelength-dependent ellipsometric parameter *Δ* offsets, which considered window birefringence effects, were varied by the optical model for data best-matching. Third, DI H_2_O was pumped into the liquid chamber by a peristaltic pump (Ismatec IPC-N 4, IDEX Health & Science GmbH), and an SE measurement was taken under liquid ambient. The substrate optical constants were allowed to vary by the optical model for data best-matching, but the differences were found to be negligible [32]. Finally, from a starting point of zero, the thickness of the Cauchy layer for BSA was allowed to vary for subsequent *in-situ* measurements.

All solutions were passed through the liquid cell using a peristaltic pump (Ismatec IPC-N 4) (Biolin Scientific) attached to Tygon tubing (Fisher Scientific) and a 90^°^ flow path 3-port valve (Hamilton) at a flow rate of 0.1 mL/min. DI H_2_O was pumped into the liquid chamber and both instruments were allowed to equilibrate for approximately 2 hr to develop a baseline reading [27]. After stabilization with DI H_2_O, BSA solution was introduced into the flow cell for 30 min and then the flow was stopped to allow the BSA molecules to adsorb to the SAM surface for approximately 70 min. DI H_2_O was then pumped into the liquid cell to remove any passively-attached BSA molecules [27]. Thickness, mass and porosity changes in SE and QCM-D induced by BSA attachment were monitored continuously, and data acquisition was performed using CompleteEASE (J.A. Woollam Co., Inc) and QSoft (Biolin Scientific) software packages for SE and QCM-D, respectively. In order to ensure measurement reproducibility for each SAM-coated Au surface, triplicate QCM-D/SE measurements for each type of SAM were acquired. QCM-D data demonstrated (data not shown) that the maximum dissipation shift was small compared to the frequency shift during the measurements. Thus, the organic thin film was assumed to be rigid and its QCM-D thickness was calculated using the Sauerbrey equation with the third frequency overtone [30, 31]. Data obtained from QCM-D/SE were statistically presented by pooling the final 30 data points of each replicate experiment for a total of 90 data points for each SAM from which adsorbed thickness, volume fraction and corresponding standard error values were determined [27]. The BSA adsorption rate over a specific period of time was calculated by dividing the areal mass of adsorbed BSA by the adsorption time [33].

To investigate BSA adsorption as a function of pH, an experiment was conducted as described above with an additional rinse step. In this experiment, after stabilization with DI H_2_O, the system was flushed with pH 2 buffer solution. A 1.0 μg/mL BSA concentration and AUT SAM were used in this experiment.

## Results

### Confirmation of SAM formation

To verify SAM coverage of the Au surfaces, an *ex-situ* measurement was done before and after SAM attachment using the spectroscopic ellipsometer and contact angle goniometer (S1 Fig). Averages SAM thickness and average water contact angles of each SAM-coated Au surface are presented in Table 2.

**Table 2:** Contact angles, SAM thickness (d_SAM_), and theoretical SAM thickness (one triplicate measurement for each SAM).

SAM	MUOH	MUA	AUT	DT10
**Contact angles (degree)**	23.37 ± 1.82	24.6 ± 0.9	47.83 ± 3.22	96.8 ± 1.1
**d**_**SAM**_ **(nm)**	0.87 ± 0.03	1.34 ± 0.02	1.74 ± 0.39	1.5 ± 0.1
**Theoretical d**_**SAM**_ **(nm)**	1.31	1.31	1.31	1.09

The length of the SAM layer can be calculated by multiplying its number of C-C bonds by the C-C length of 0.154 nm and by taking into account the C-C bond angle of 109.5^°^. The expected SAM thickness was determined by multiplying the calculated SAM length by *sin*(60^°^) [31]. The contact angles of MUOH, MUA, and AUT were all less than 90^°^ while that of DT10 was greater than 90^°^, indicating the formation of hydrophilic-terminal and hydrophobic-terminal SAMs, respectively [34, 35]. SAM adsorption on Au surfaces has been investigated in a number of previous studies with inconsistent results [2, 36, 37]. In a previous study, static contact angles of MUOH, MUA, and DT10 were reported as 10°, 15^°^ and 97^°^, respectively [2], while those of MUA and DT10 were reported as ≤ 10^°^ and ~ 110^°^, respectively, in another study [36]. The discrepancy in reported contact angles of MUOH, MUA, and DT10 were also observed in a previous study, which might indicate that the yield of SAMs was incomplete as monolayer formation depends on the immersion time, the purity of the thiols, and the quality of the Au surface [2].

The measured SAM thicknesses corresponded to the estimated SAM thickness for MUA and AUT SAMs (Table 2). The thickness of DT10 observed in this study is in agreement with a prior study by Mendoza *et*. *al*. [36]. The combination of contact angle and *ex-situ* measurement data demonstrates the existence of near-complete coverage of hydrophobic SAMs (contact angles > 90^°^) and hydrophilic SAMs (contact angle < 90^°^) on the Au surfaces used in this study; however, we cannot exclude the possibility that a discontinuous monolayer was formed for all SAMs used in this study.

### *In-situ* thickness and porosity of BSA deposited thin film on different SAMs

**Fig 1:**
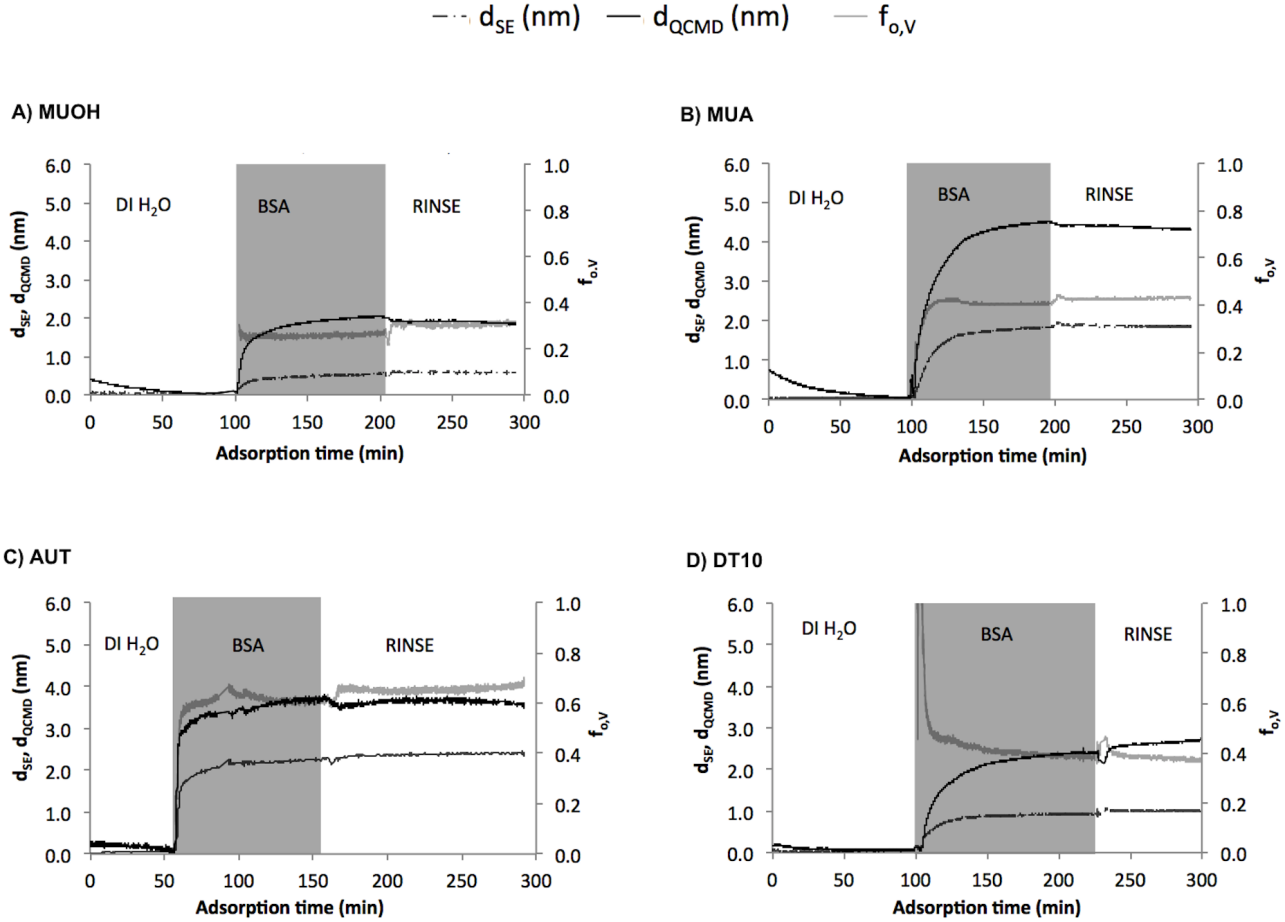
*In-situ* SE (d_SE_), QCM-D (d_QCMD_) thickness and adsorbate volume fraction (f_o,V_) on various SAM surfaces. (A) MUOH; (B) MUA; (C) AUT; and (D) DT10.

**Fig 2:**
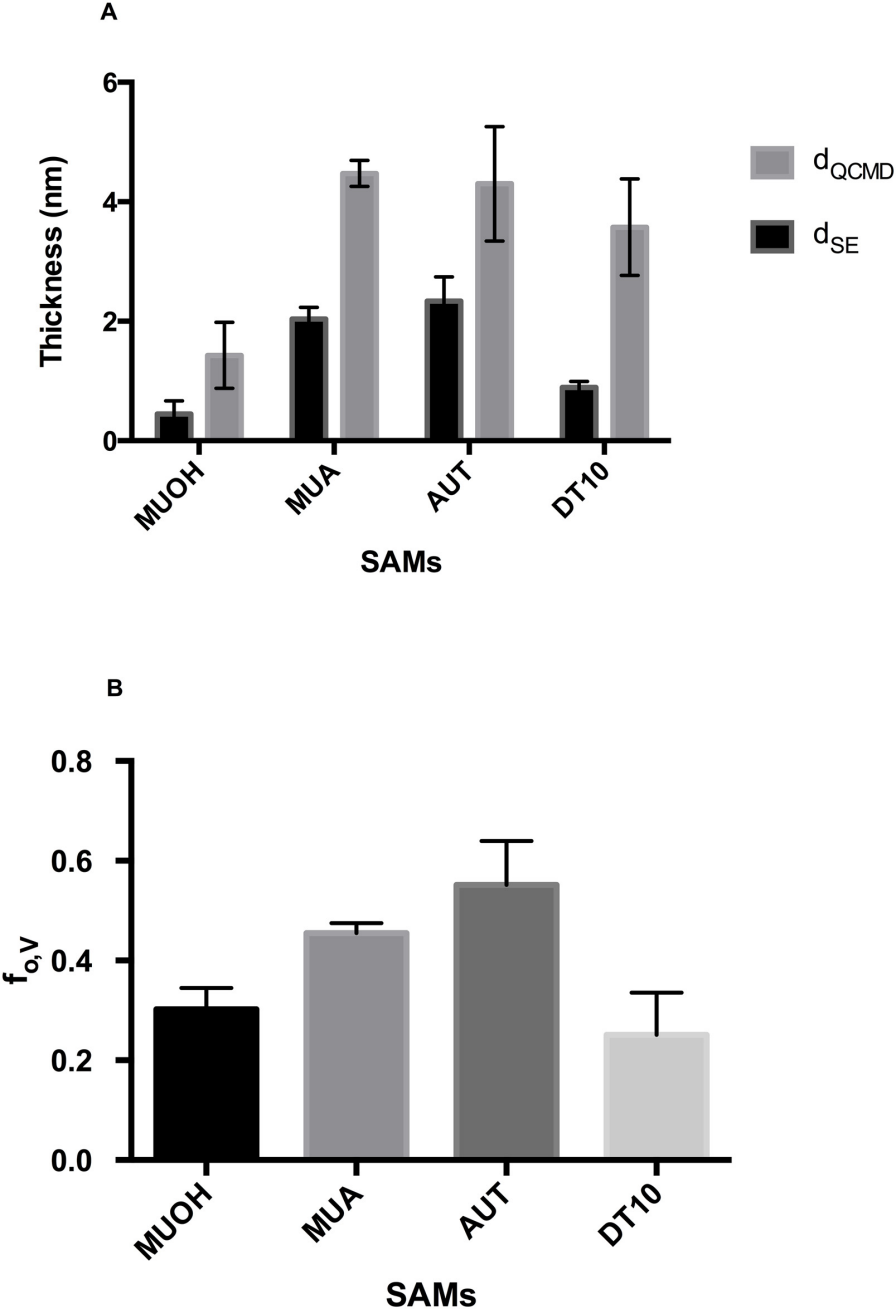
Averages from triplicate SE/QCM-D measurements. (A) SE and QCM-D thickness parameters (d_SE_, d_QCMD_, respectively) of different SAM surfaces with associated standard errors. (B) Adsorbate volume fractions (f_o,V_) of different SAM surfaces with associated standard errors.

Representative *in-situ* QCM-D/SE measurements of BSA adsorption are presented in Fig 1. d_SE_ only represents the contribution of adsorbate while d_QCMD_ represents both adsorbate and associated solvent entrapped by the adsorbate layer [30–32, 38, 39]. The combination of these two parameters allows for determination of an additional parameter, the adsorbate volume fraction (f_o,V_ = d_SE_/d_QCMD_). The volume fraction quantitatively describes the porosity of thin films [30–32, 38, 39] with increasing volume fraction indicative of decreasing porosity (i.e. less ambient solvent inclusion) [27]. A summary of changes in absorbed thickness and volume fraction for *in-situ* QCM-D/SE measurements is presented in Fig 2. The thickness presented in Panel A is the difference in adsorbed BSA upon rinsing with DI H_2_O relative to the measured thickness prior to BSA adsorption.

BSA adsorption occurred on all SAM surfaces and, as expected, the immobilized BSA thickness varied across different SAMs (Fig 1). The lowest amount of BSA attachment was measured on the neutral hydrophilic MUOH surface (Fig 1A), with d_SE_ = (0.450±0.019) nm (95% CI [0.41, 0.49]). BSA attachment to the neutral hydrophobic DT10 surface was larger with a measured d_SE_ = (0.891±0.009) nm (95% CI [0.87, 0.91]). For charged surfaces, BSA adsorption to MUA (Fig 1B), a negatively charged hydrophilic surface, was comparable to adsorption to AUT (Fig 1C), a positively charged hydrophilic surface as measured by SE. For charged surfaces, BSA adsorption was approximately 4.5–5.2 and 2.3–2.6 times greater when compared with adsorption to the neutral MUOH and DT10 surfaces, respectively.

The thickness of the adsorbed BSA layer as determined by QCM-D on the neutral hydrophilic MUOH surface was d_QCMD_ = (1.430±0.048) nm. BSA adsorption to neutral hydrophobic DT10 surface was 2.5 times greater than to the MUOH surface (Fig 1A and 1D). BSA adsorption to charged surfaces was observed to be 3.0 and 1.2 times greater than BSA attachment onto neutral MUOH and DT10 surfaces, respectively (Fig 1). BSA adsorption to the charged hydrophilic AUT and MUA surfaces was comparable (S1 Table). Results from QCM-D/SE indicate that BSA adsorption increases as MUOH < DT10 < MUA < AUT.

Data on the volume fraction of the attached BSA proteins indicates SAM surface properties influence the packing arrangement of surface-associated BSA protein (Fig 2). The volume fraction of BSA proteins attached to MUOH and DT10 surfaces were similar with f_o,V_ for MUOH = 0.303±0.045 (95% CI [0.29, 0.31]) and f_o,V_ for DT10 = 0.251±0.009 (95% CI [0.23, 0.27]), indicating that BSA molecules likely had similar arrangement on these surfaces or BSA adsorption on theses surfaces had similar porosity. The BSA volume fraction (f_o,V_ = 0.552±0.009 (95% CI [0.53, 0.57])) was highest on the positively-charged hydrophilic AUT surface, followed by the negatively-charged hydrophilic MUA surface (f_o,V_ = 0.477±0.002 (95% CI [0.45, 0.46])).

### Areal mass BSA and number of deposited BSA molecules on different SAMs

**Fig 3:**
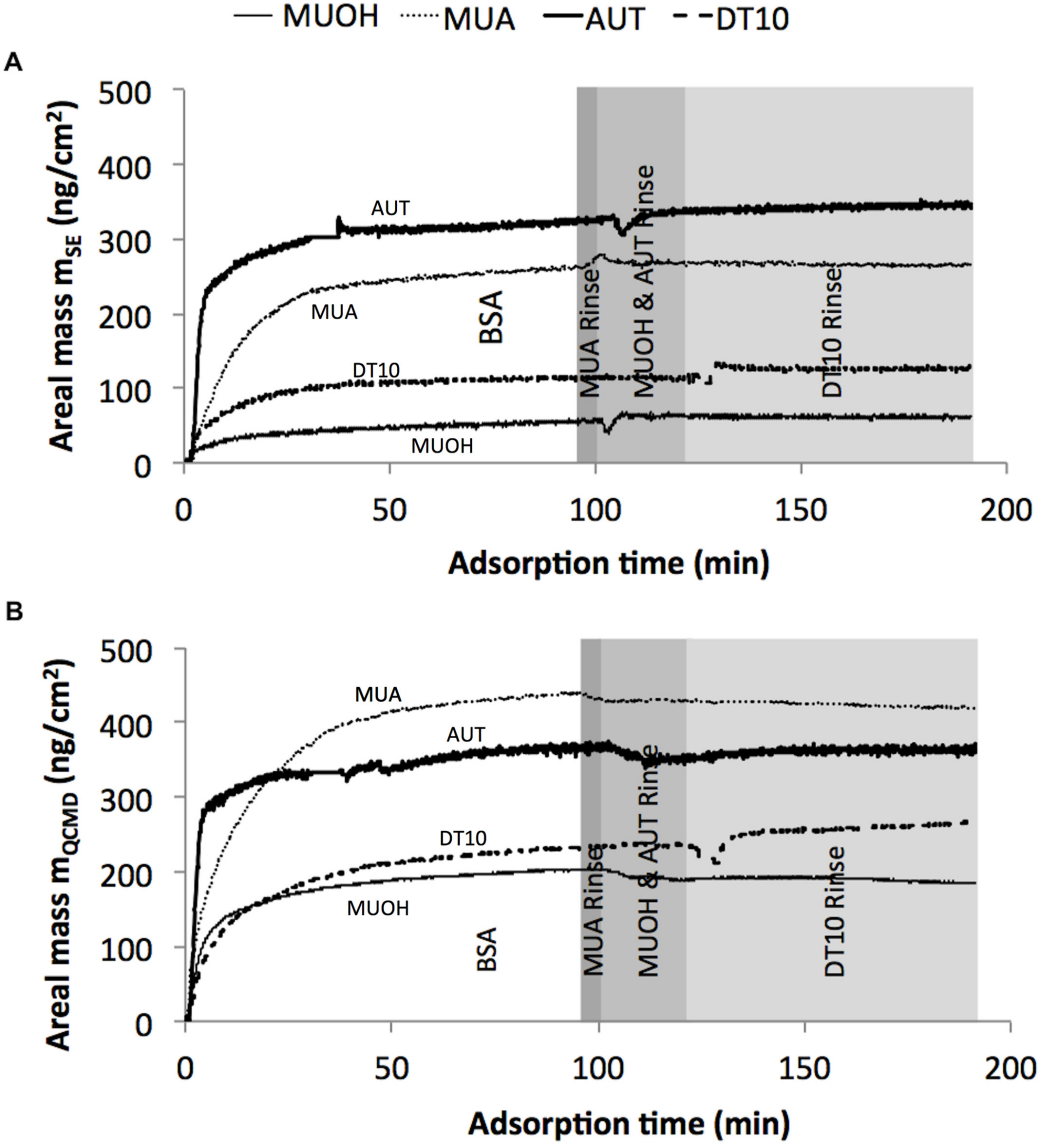
BSA areal mass attached on different SAM-coated Au surfaces measured by SE and QCM-D. (A) m_SE_: areal mass measured by SE, and (B) m_QCMD_: areal mass measured by QCM-D. Time 0 was the start of BSA phase following initial DI H_2_O phase. Different shades represent associated rinse phase of each SAM. The rinse phases of all measurement were not simultaneous.

Fig 3 shows the calculated areal mass of BSA attached to each of the four different SAMs surfaces. “Dry mass” (m_SE_, Fig 3A) and “wet mass” (m_QCMD_, Fig 3B) show similar shapes, and m_QCMD_ of a given SAM is always higher as the measured mass includes the liquid solvent. Although the BSA equilibration periods were slightly different for each SAM evaluated, the ultimate BSA mass adsorbed was not influenced as maximum adsorption was reached prior to the DI H_2_O rinse phases.

Using the ‘dry mass’ of BSA attached to the surface, 2.77 x 10^12^–3.17 x 10^12^ BSA molecules were deposited on MUA and AUT and represented 2.3–5.2 times the number of BSA molecules deposited on DT10 and MUOH, respectively (S1 Table). Similar to trends observed for d_SE_ and d_QCMD_, the wet and dry mass deposited were highest on MUA and AUT, and were lower on DT10 and MUOH. The trend in mass deposited was MUOH < DT10 < MUA ≤ AUT (S1 Table).

### Interaction of BSA on AUT SAM as a function of BSA solution concentration

**Fig 4:**
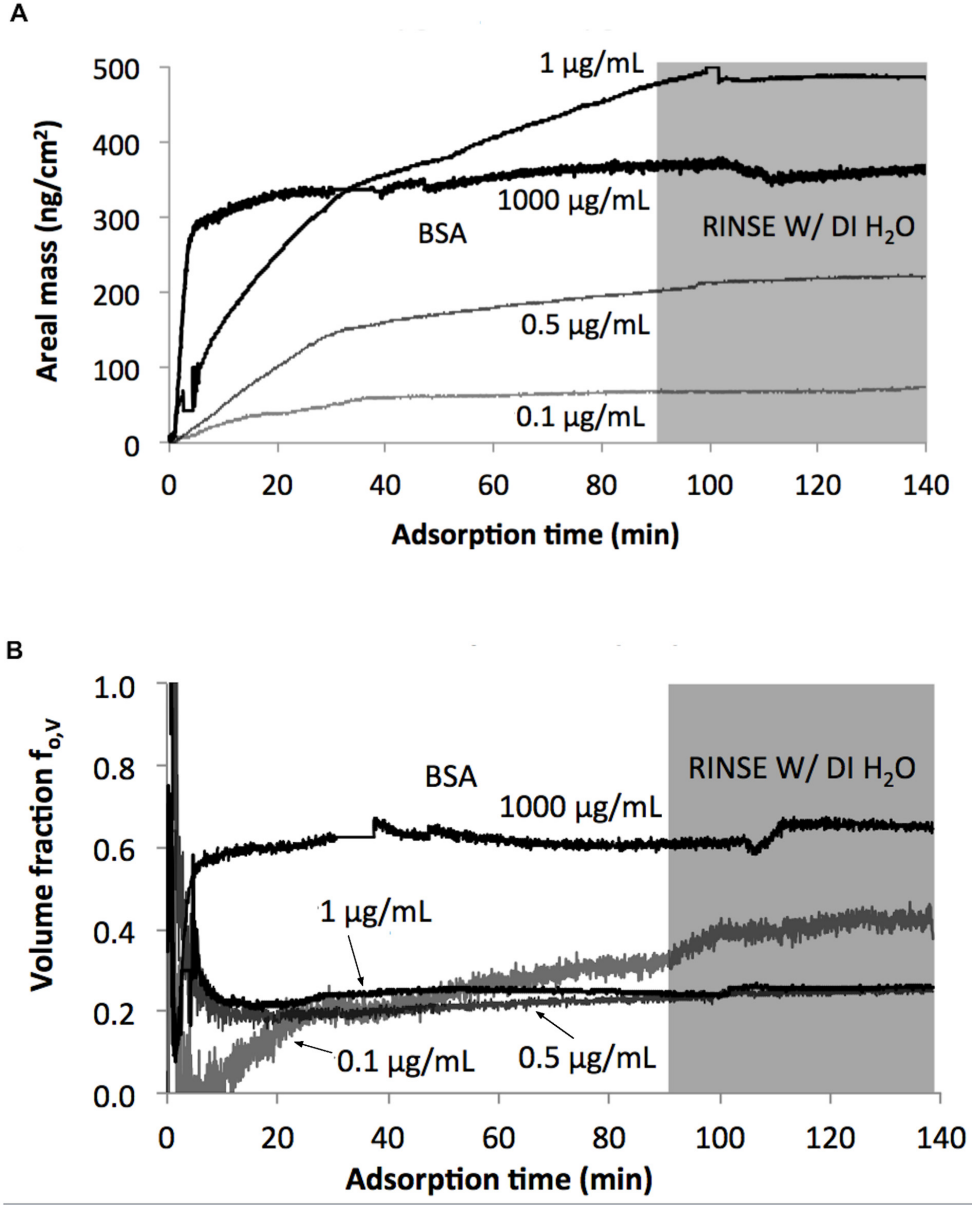
Interaction of BSA on AUT SAM as a function of BSA concentrations. (A) Areal mass of BSA attachment detected by QCM-D. (B) Volumetric fraction of BSA attachment. Time 0 was the start of BSA phase following initial DI H_2_O phase.

Decreasing BSA concentration from 1.0 μg/mL to 0.1 μg/mL generally resulted in decreased protein adsorption (Fig 4). The average areal mass from triplicate measurements using the 1,000 μg/mL BSA solution was (0.46±0.008) μg/cm^2^ (S1 Table) which is similar to the area mass of 0.47 μg/cm^2^ measured for the 1.0 μg/mL BSA solution concentration. At a solution concentration lower than 0.1 μg/mL, the adsorption was not detected (data not shown), therefore, the limit of combinatorial *in-situ* QCM-D/SE detection for BSA occurs at aqueous concentrations between 0.1 and 1.0 μg/mL BSA, highlighting the sensitivity of this instrument.

Although the adsorbed protein was comparable at both 1,000 μg/mL and 1.0 μg/mL concentrations, their respective porosities were not similar (Fig 4B). At 1,000 μg/mL, the volume fraction of BSA was approximately 0.55±0.009 (S1 Table) and 10–25% higher than the volume fractions determined for the 1.0 μg/mL BSA solution concentration. Higher BSA concentration resulted in more densely packed BSA molecules.

### BSA adsorption rate as a function of surface properties and of BSA concentration

**Fig 5:**
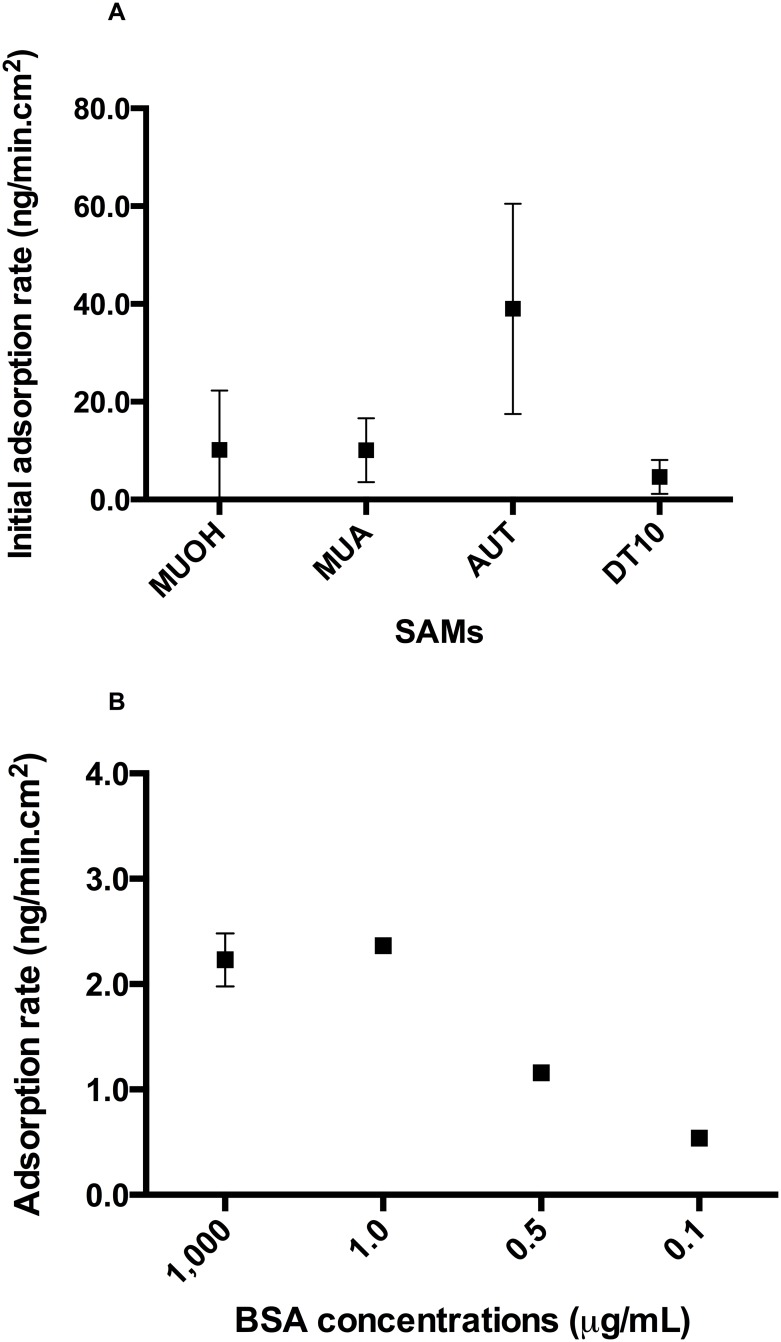
BSA adsorption rate measurements. (A) BSA initial adsorption rate and (B) BSA overall adsorption rate as a function of surface properties. The error bars represent standard deviations.

Adsorption kinetics are important in applications such as organic foulants adsorption onto clean membranes [40]. Fig 5 represents the initial BSA adsorption rates, determined from the linear section of the kinetic curves, and overall BSA adsorption rates, determined from measurements when the system was at equilibrium, as a function of SAM surfaces properties. The BSA adsorption to surfaces can be observed as a two-phase process which the first phase represented rapid deposition (represented as initial adsorption rate) of BSA followed by a second phase with slower adsorption (Fig 1) [31]. The SAM surfaces strongly affected the BSA adsorption rate. The initial adsorption rate was observed to be highest on the AUT surface ((39.0±10.1) ng/min.cm^2^ (95% CI [-14.41, 92.43])), which was four times greater than adsorption to the MUOH and MUA surfaces; and ten times greater than adsorption to the DT10 surface (Fig 5A). The trend in overall adsorption rate was different from the initial adsorption rate as MUA > AUT > MUOH > DT10 (Fig 5B).

### Influence of pH on the adsorption of BSA to AUT SAM

**Fig 6:**
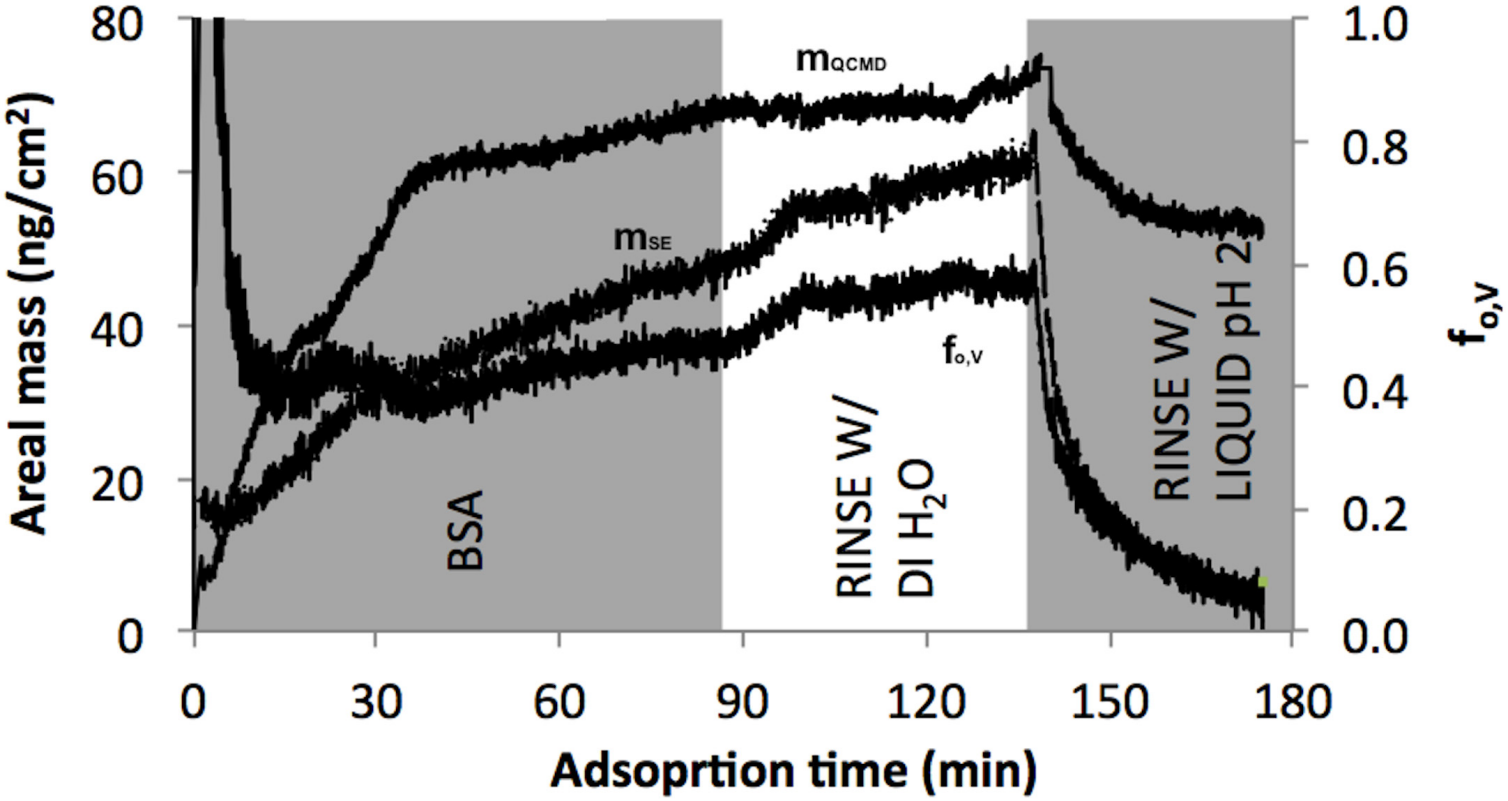
Influence of pH solution on the adsorption of BSA to AUT SAM. pH 2 solution was flushed into the liquid cell immediately preceding DI H_2_O rinsing phase. Time 0 was the start of BSA phase following initial DI H_2_O phase which was not shown.

The areal mass of adsorbed BSA decreased after approximately 40 min of flushing with pH 2 solution (Fig 6). After the pH 2 rinsing phase, it is likely that all the adsorbed BSA molecules were detached from the surface yielding a nearly zero volume fraction. An areal mass of approximately 50 ng/cm^2^ was detected by QCM-D after the pH 2 solution rinsing phase and represented the amount of solvent only.

## Discussion

In this study, we demonstrate BSA adsorption to selected alkanethiol SAMs, which represent various types of environmentally-relevant surfaces and included variability in surface charge and hydrophobicity.

### BSA interaction with surfaces as a function of surface properties

Because the SAMs investigated in this study had similar carbon chain lengths (Table 1), we can attribute differences in BSA adsorption to differences between SAM functional tail groups. The highest adsorbed thickness occurred on the charged, hydrophilic MUA and AUT surfaces (i.e. carboxyl and amine groups, respectively). Meanwhile neutral surfaces such as DT10 and MUOH had lower BSA adsorption thicknesses, in which the hydrophilic tail group (hydroxyl group) attracted fewer BSA molecules than the hydrophobic one (methyl group). The areal adsorbed mass exhibited a similar trend as the adsorbed thicknesses (S1 Table). This finding was also observed in previous research in which the absorbed mass decreased as the surfaces became more hydrophilic [1, 12, 14, 40–43]. The predominant mechanism attributed to BSA adsorption to DT10 is the hydrophobic interactions between the methyl group and the hydrophobic domains of BSA [42, 43]. The predominant mechanism likely responsible for BSA adsorption to hydroxyl-terminated MUOH is the hydrogen-bonding interactions between the terminal alcohol and the protein [40, 42–44].

For the charged surfaces, since both negatively and positively charged MUA and AUT surfaces exhibited BSA adsorption at neutral pH, electrostatic interaction is not likely to be the predominant adsorption mechanism [43]. At neutral pH, the BSA proteins are negatively charged and are repelled from the negatively charged MUA surface and a decrease in BSA adsorption are expected, however, the adsorption of BSA proteins to negatively charged surfaces in aqueous solution at neutral pH was observed (Fig 1B). The adsorption of BSA proteins to negatively charged surface at neutral pH was also observed in previous studies [18, 40, 42]. The adsorption to the MUA surface in other studies may be explained by the presence of positively charged domains (lysine, histidine) on the BSA surface. In a previous study [43], Human Serum Albumin (HSA) attachment to negatively charged surfaces was observed at neutral pH. An increase in pK_a_ of the carboxylic functional group when adsorbed in a tightly packed SAM surface can lead to the protonation of the carboxylic-terminated groups at neutral pH, resulting in the strong hydrogen bonding interactions with the BSA proteins [43, 45, 46]. The exposure of the hydrophobic regions of the alkyl chains due to the disordered MUA SAMs could contribute BSA adsorption to this surface [43]. The large BSA adsorption to positively charged surface can be attributed to the strong electrostatic interactions between the AUT surface and BSA proteins.

Among all SAMs investigated, MUOH exhibited the least adsorption of BSA, which is in agreement with a prior study evaluating BSA adsorption to OH-terminal surfaces [40, 41]. BSA adsorption to MUOH was (0.143±0.005) μg/cm^2^ (S1 Table), which compares well with a value of 0.19 μg/cm^2^ reported in Frida’s study of BSA adsorption to MUOH on Au after 50 min [2]. However, the BSA adsorption process in Frida’s study continues to level out after 50 min while the BSA adsorption to MUOH in this study saturated after 1 min of BSA introduction to the liquid cell (Fig 3B) [2]. From this study, it was observed that the quantity of BSA adsorption was found to decrease in the order –NH_2_ ≥ –COOH > –CH_3_ > –OH, which agrees with a previous study where positively-charged SAMs surface exhibited a greater amount of BSA adsorption than negatively-charged surfaces and greater BSA adsorption was observed on hydrophobic surfaces [40, 42].

Charged surfaces had greater BSA packing density when compared with neutral surfaces (Fig 2B). This finding is consistent with a previous study demonstrated MUA SAMs form a compact monolayer while DT10 SAMs present largely uniform but less compact areas in scanning tunneling microscopy images [36]. The densely-packed monolayer of MUA compared to DT10 is likely the reason leading to a higher density of BSA molecules on MUA given that more binding sites are available. A similar explanation can be applied for amine- and hydroxyl- terminated alkanethiols however more research is necessary. In general, Fig 2B implies that BSA proteins are loosely packed on neutral surfaces while increasing packing occurs on charged surfaces.

The measured thickness (d_SE_ and d_QCMD_) of BSA adsorption to MUOH and DT10 are not directly proportional to the volume fraction (Fig 2). Although DT10 has a higher d_SE_, more ambient solvent was entrapped within the attached proteins leading to higher d_QCMD_. For BSA adsorption to MUA and AUT, it is likely that during the adsorption process, BSA adsorbed to MUA substrate entrapped more ambient solvent compared to AUT leading to observation of a lower volume fraction on MUA.

**Fig 7:**
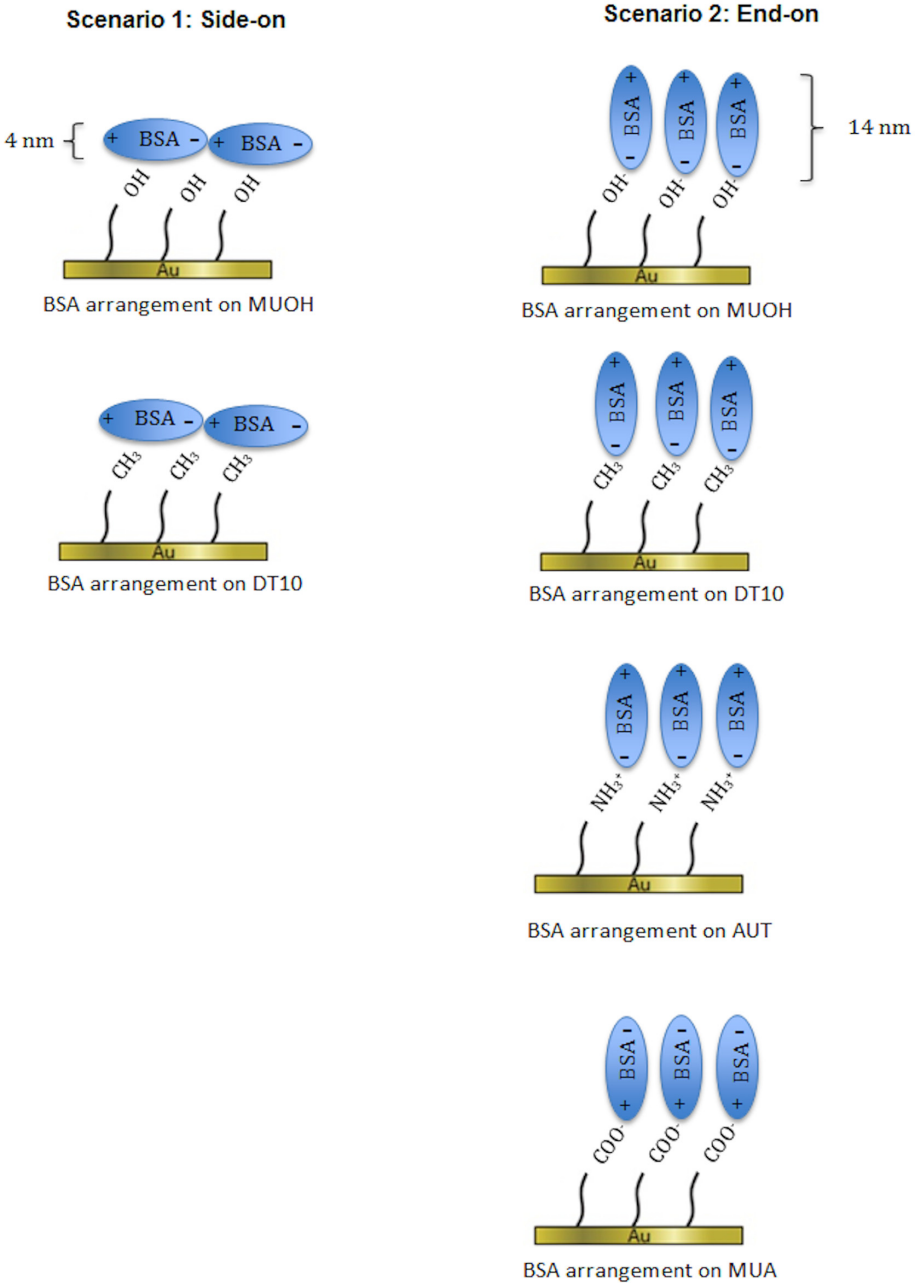
BSA arrangement scenarios on various SAM surfaces. Left: Scenario 1 –Side-on; Right: Scenario 2: End-on.

Due to its three dimensional shape, BSA molecules may align in side-on or end-on arrangements [14, 21] (Fig 7). BSA has the approximate dimensions 4 nm x 4 nm x 14 nm in aqueous solution [29], therefore the thickness of a BSA monolayer should be 4 nm in a side-on scenario and 14 nm in an end-on scenario. For the side-on scenario, a BSA molecule occupies an area of approximately 56 nm^2^ resulting in a maximal density of 2.75 x 10^12^ BSA molecules per sensor given that the sensor area available for attachment is 154 mm^2^. For the end-on scenario, a BSA molecule occupies an area of 16 nm^2^ corresponding to 9.63 x10^12^ BSA molecules per sensor. It was observed that the average number of deposited BSA molecules measured on MUA and AUT SAM surfaces were slightly greater than the maximum number of BSA molecules in the side-on scenario and approximately three times lower in the end-on scenario (S1 Table). It should be noted that QCM-D/SE detects the average thickness of the thin film on the Au surface. Therefore, if the SAMs were not ideally packed, for instance with SAM patches oriented at different rotations or contaminants blocking potential chemisorption sites [31], BSA molecules attachment would be incomplete leading to lower than expected values of d_QCMD_. Additionally, since the measured thickness of the BSA adsorption layer was greater than 4.0 nm (Fig 7 and S1 Table), the end-on scenario may be a likely arrangement for BSA adsorption to MUA and AUT. For MUOH and DT10 surfaces, both scenarios are possible (Fig 7) as the maximum number of BSA molecules were 2–16 times greater than the measured number of BSA molecules and the measured thickness was lower than 4.0 nm (Fig 7 and S1 Table). The BSA arrangement scenarios as a monolayer are further supported by a previous study that BSA aggregation was not observed (no expansion of BSA hydrodynamic diameter) at BSA concentration of 1.0 mg/mL at room temperature [5].

### BSA adsorption rate as a function of surface properties and of BSA concentration

Two-phase BSA adsorption on SAMs surfaces in aqueous solution was also observed in previous studies [6, 27, 31, 41]. The first phase occurred as BSA solution was initially in contact with excess sorption sites on SAM. The second phase was then slower until it reached a plateau as most binding sites are occupied in the first phase and the monolayer uniformity is improved by the molecule ordering and packing in this phase [31]. The BSA initial adsorption rate was greatest on the positively-charged hydrophilic AUT surface (Fig 5A). The lowest affinity was towards the neutral hydrophobic DT10 surface (Fig 5A). The trend in intial adsorption rate is consistent with the trend of both volume fraction and adsorbed areal mass (S1 Table). It can be implied that the electrostatic interactions responsible for BSA adsorption to positively charged AUT surfaces are stronger than the hydrophobic interaction between BSA and hydrophobic DT10 surface.

The overall BSA adsorption rate to neutral MUOH and DT10 surfaces were comparable and were lower than the overall BSA adsorption rate to charged AUT and MUA surfaces. These results suggest that higher initial BSA adsorption rates occur on –NH_2_ and –COOH surfaces, which is supported by a previous finding [40].

Despite comparable adsorbed areal mass of BSA at equilibrium on AUT and MUA (S1 Table), initial adsorption rates were markedly higher for AUT than MUA (Fig 5) suggesting that SAM type could be a controlling component in the initial adsorption phase and consequently play a critical role in monolayer uniformity.

### BSA interaction with a surface as a function of BSA concentrations

BSA adsorption increased with increasing protein concentration in the range of 0.1–1.0 μg/mL. BSA solution concentrations of 1.0 μg/mL and 1,000 μg/mL yielded no difference in adsorbed BSA, indicating that BSA adsorption was at saturation (S1 Table). At concentrations lower than 0.1 μg/mL, no adsorption could be detected by the combinatorial *in-situ* QCM-D/SE. We observed that at high BSA concentrations (1,000 μg/mL), a higher volume fraction was measured when compared to lower concentrations (0.1–1.0 μg/mL) (Fig 4B). The smaller number of BSA molecules resulted in looser packing on the SAM surface. When more BSA molecules were available in solution, more binding sites were occupied leading to a higher adsorbate volume fraction.

### Influence of pH on BSA adsorption

BSA adsorption/desorption to AUT was influenced by solution pH. Since BSA is negatively charged at neutral pH, BSA molecules would electrostatically adsorb to the positively charged AUT SAM. Inversely, the electrostatic repulsion between positively charged AUT substrate and positively charged BSA may prevent the binding of protein to AUT surface at pH 2, leading to BSA desorption [6] (Fig 6). At pH 2, BSA molecules fold and expose the hydrophobic portions of the molecules to the AUT surface leading to desorption from the surface. Since the adsorbed BSA molecules were completely removed after the pH 2 rinsing phase (f_o,V_ ~ 0), we believe that the positive QCM-D signal can be attributed to the desorption of BSA molecules on AUT surface.

## Conclusions

Kinetic adsorption of BSA to various SAM surfaces were evaluated using *in-situ* combinatorial QCM-D/SE. Adsorption of BSA varied as a function of surface properties, BSA concentrations and pH. BSA adsorption to the investigated SAMs is a two-phase process in which the initial adsorption rate is greatest for the positively-charged hydrophilic AUT surface and smallest for the neutral hydrophobic DT10 surface. A greater quantity of BSA molecules, a larger thickness of the BSA thin film layer, and a more densely-packed BSA molecules occur on charged surfaces when compared to neutral surfaces. Both negatively and positively charged surfaces have similar adsorption in terms of the film thickness, the quantity of adsorbed BSA, and the thin film porosity. However, the initial kinetic adsorption rate of BSA for positively charged surface is faster than for negatively charged surfaces. The density of adsorbed BSA depends on the molecular structure of the free-tail functional group of SAM, which was observed as highly packed for –COOH and –NH_2_, and less densely packed for –CH_3_ and –OH. Hydrophobic interactions and hydrogen bonding are responsible for BSA adsorption to hydrophobic DT10 and neutral MUOH surfaces, respectively, while the combination of electrostatic and hydrophobic interactions are involved in BSA adsorption to charged AUT and MUA surfaces. The quantity of adsorbed BSA molecules to AUT surface increased under increasing BSA concentrations. It was confirmed in the study that the adsorbed BSA molecules desorbed at aqueous pH lower than its IEP.

## Supporting Information

S1 Fig Contact angles measurements of Au surfaces before and after coated with SAMs. (A) MUOH, (B) MUA, (C) AUT, and (D) DT10.
(TIF)

S2 Fig BSA thickness on AUT-coated Au surface as a function of BSA concentrations. d_SE_: SE thickness, d_QCMD_: QCM-D thickness, and f_o,V_: adsorbate volume fraction. (A) 1.0 μg/mL with additional rinse phase with pH 2 following DI water rinse phase; (B) 0.5 μg/mL; and (C) 0.1 μg/mL.
(TIF)

S3 Fig BSA areal mass on AUT-coated Au surface as a function of BSA concentrations. (A) 1.0 μg/mL with an additional rinse phase with pH 2 following with DI water rinse phase; (B) 0.5 μg/mL; and (C) 0.1 μg/mL.
(TIF)

S1 Table Summary of measured and calculated results with corresponding standard errors. d_SE_: SE thickness; d_QCMD_: QCM-D thickness; Δm_SE_: SE adsorbate areal mass changes; Δm_QCMD_: QCM-D adsorbate areal mass changes; and f_o,V_: adsorbate volume fraction.
(PDF)
