# Supplementary figures and images for: Investigation of Bovine Serum Albumin (BSA) Attachment onto Self-Assembled Monolayers (SAMs) Using Combinatorial Quartz Crystal Microbalance with Dissipation (QCM-D) and Spectroscopic Ellipsometry (SE)
Source: PLoS One. 2015 Oct 27;10(10):e0141282. doi: 10.1371/journal.pone.0141282 (PMC4624694; doi:10.1371/journal.pone.0141282)

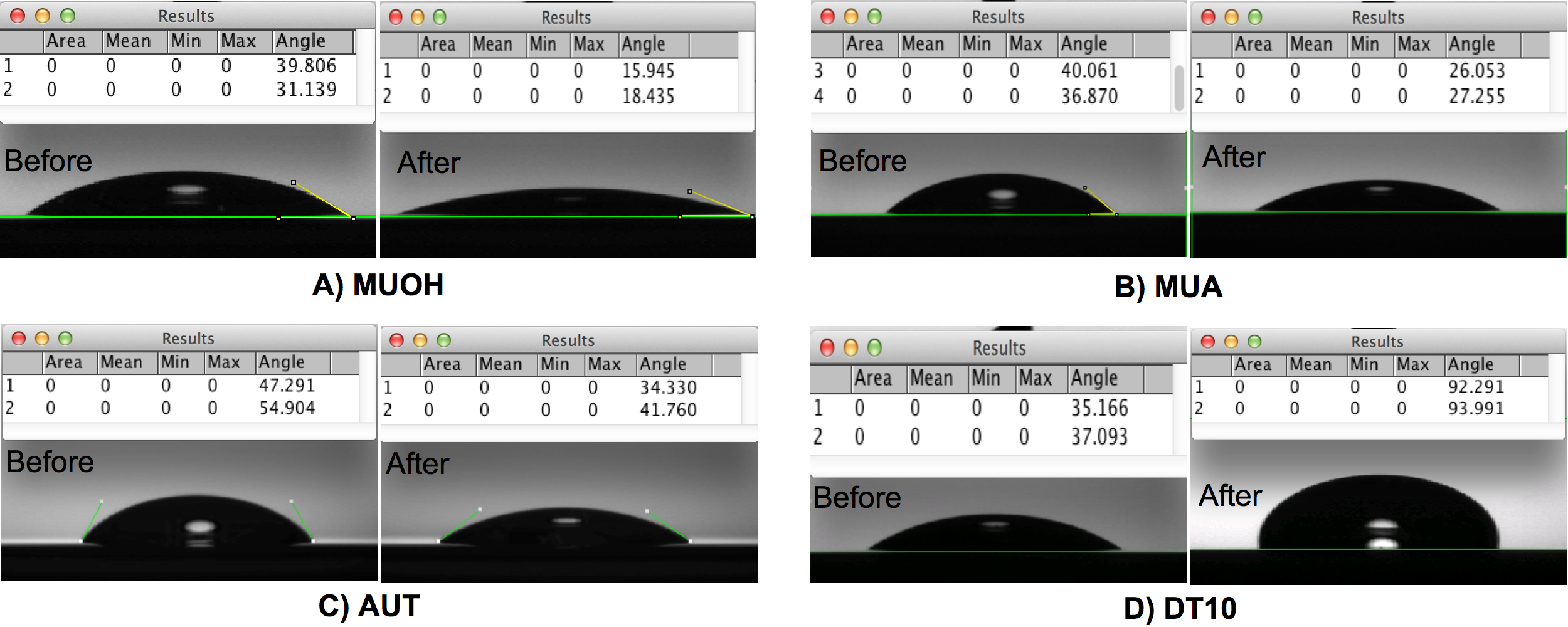

Supplement: S1 Fig — (A) MUOH, (B) MUA, (C) AUT, and (D) DT10. (TIF) [file pone.0141282.s001.tif]

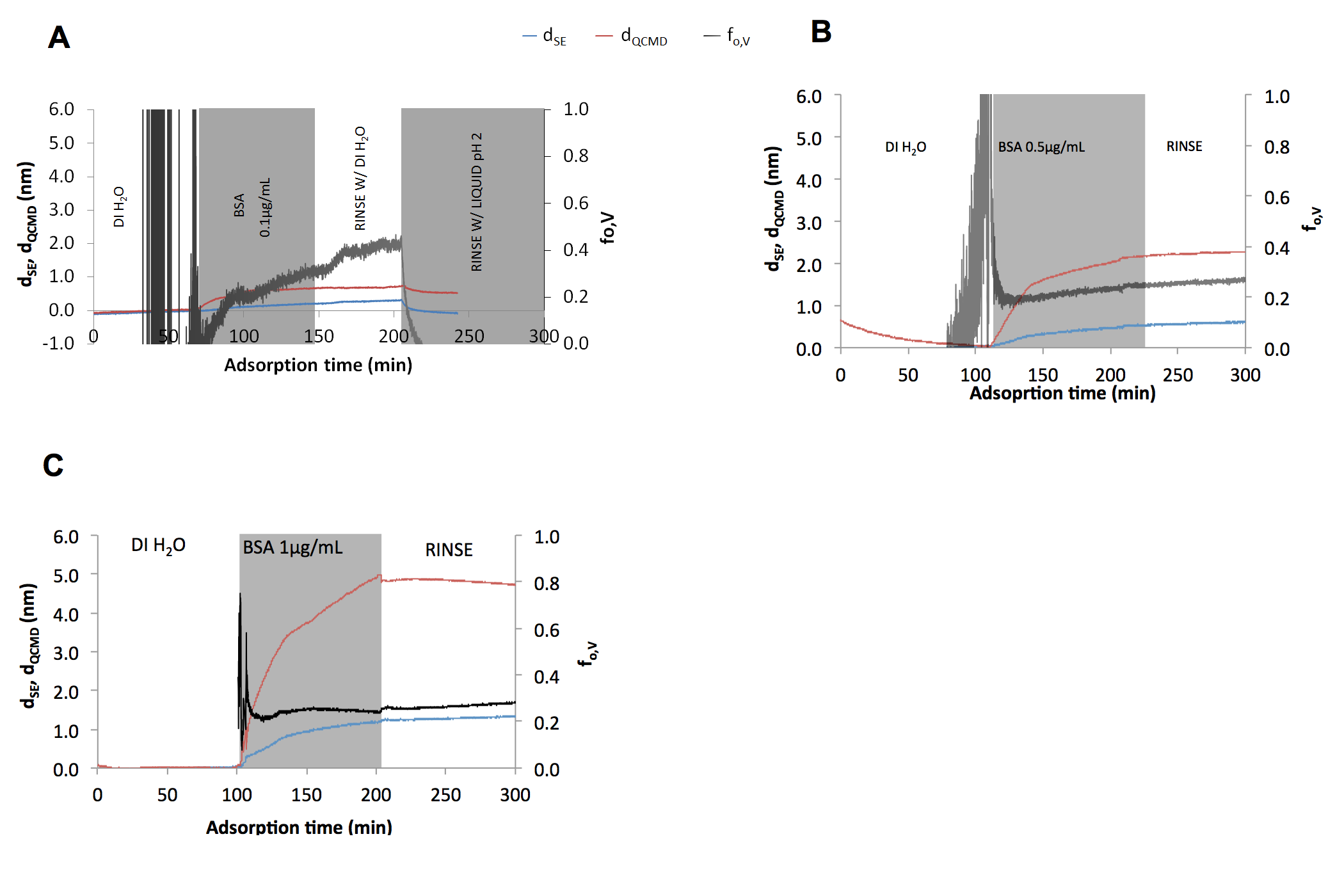

Supplement: S2 Fig — dSE: SE thickness, dQCMD: QCM-D thickness, and fo,V: adsorbate volume fraction. (A) 1.0 μg/mL with additional rinse phase with pH 2 following DI water rinse phase; (B) 0.5 μg/mL; and (C) 0.1 μg/mL. (TIF) [file pone.0141282.s002.tif]

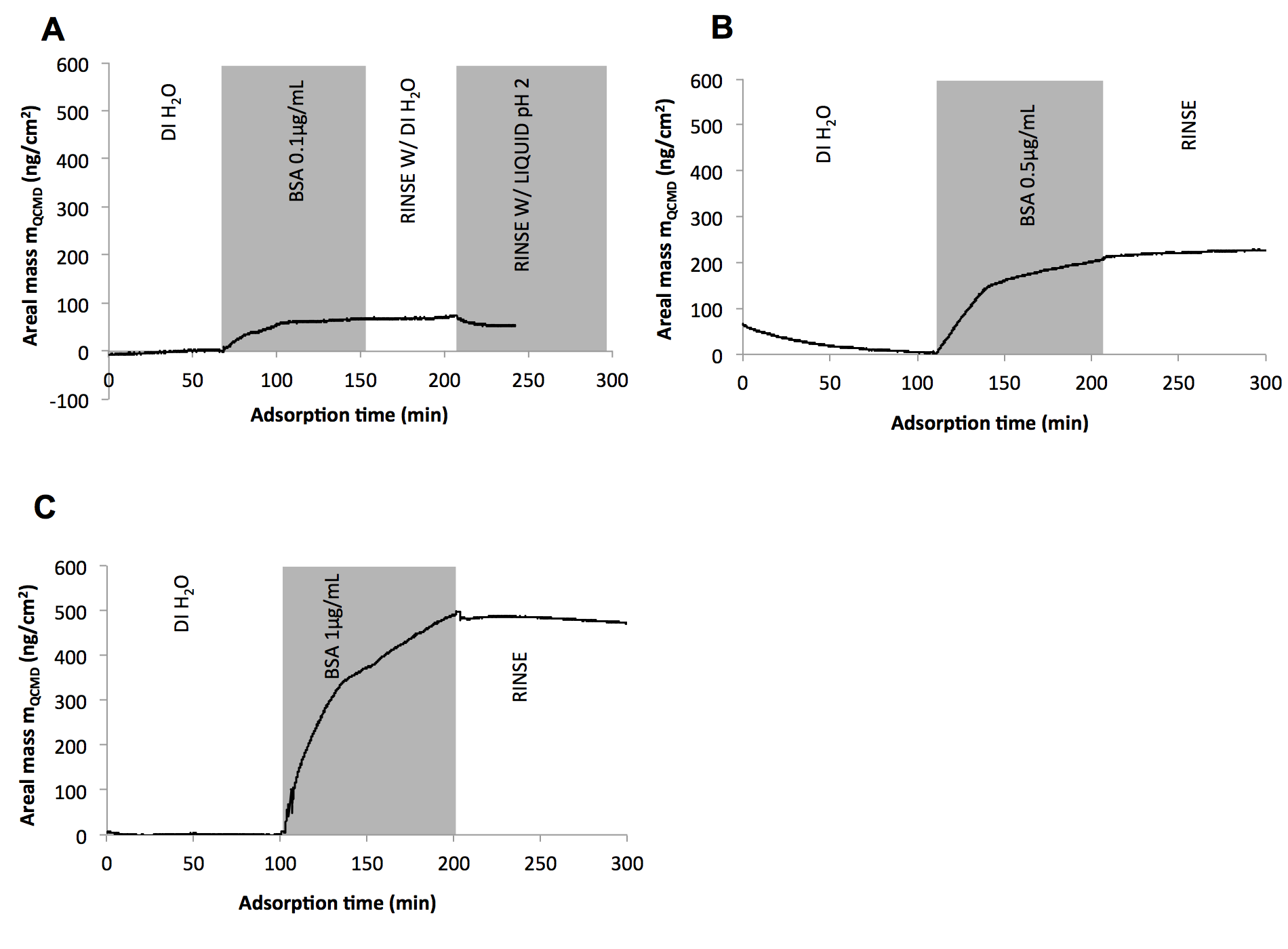

Supplement: S3 Fig — (A) 1.0 μg/mL with an additional rinse phase with pH 2 following with DI water rinse phase; (B) 0.5 μg/mL; and (C) 0.1 μg/mL. (TIF) [file pone.0141282.s003.tif]
