# Supplementary material for: Investigation of Bovine Serum Albumin (BSA) Attachment onto Self-Assembled Monolayers (SAMs) Using Combinatorial Quartz Crystal Microbalance with Dissipation (QCM-D) and Spectroscopic Ellipsometry (SE)
Source: PLoS One. 2015 Oct 27;10(10):e0141282. doi: 10.1371/journal.pone.0141282 (PMC4624694; doi:10.1371/journal.pone.0141282)
Supplement: S1 Table — dSE: SE thickness; dQCMD: QCM-D thickness; ΔmSE: SE adsorbate areal mass changes; ΔmQCMD: QCM-D adsorbate areal mass changes; and fo,V: adsorbate volume fraction. (PDF) [file pone.0141282.s004.pdf]

Supporting information

S1 Table: Summary of measured and calculated results with corresponding standard errors. d_{SE}: SE thickness; d_{QCMD}: QCM-D thickness; Δm_{SE}: SE adsorbate areal mass changes; Δm_{QCMD}: QCM-D adsorbate areal mass changes; and $f_{o,V}$: adsorbate volume fraction.

SAM-coated surfaces	d_{SE} (nm)	d_{QCMD} (nm)	Δm_{SE} (µg/cm^2)	Δm_{QCMD} (µg/cm^2)	$f_{o,V}$	# of BSA molecules per cm^2
**MUOH**	0.450±0.019	1.430±0.048	0.067±0.003	0.143±0.005	0.303±0.045	6.07×10^{11}
**MUA**	2.039±0.017	4.475±0.019	0.306±0.003	0.447±0.002	0.455±0.002	2.77×10^{12}
**AUT**	2.335±0.035	4.300±0.083	0.350±0.005	0.456±0.008	0.552±0.009	3.17×10^{12}
**DT10**	0.891±0.009	3.574±0.070	0.134±0.001	0.357±0.007	0.251±0.009	1.21×10^{12}
